# The Role of Microglia in the (Mal)adaptive Response to Traumatic Experience in an Animal Model of PTSD

**DOI:** 10.3390/ijms23137185

**Published:** 2022-06-28

**Authors:** Kesem Nahum, Doron Todder, Joseph Zohar, Hagit Cohen

**Affiliations:** 1 Department of Psychology Experimental Psychology, Brain and Cognition, Faculty of Humanities and Social Sciences, Ben-Gurion University of the Negev, Beer-Sheva 8410501, Israel; kesem.nah@gmail.com; 2 Beer-Sheva Mental Health Center, Ministry of Health, Anxiety and Stress Research Unit, Faculty of Health Sciences, Ben-Gurion University of the Negev, Beer-Sheva 8461144, Israel; doron.todder1@pbsh.health.gov.il; 3 Post-Trauma Center, Sheba Medical Center, Tel Aviv University, Tel Aviv 52621, Israel; jozohar@gmail.com; 4 Department of Clinical Biochemistry and Pharmacology, Faculty of Health Sciences, Ben-Gurion University of the Negev, Beer-Sheva 8410501, Israel

**Keywords:** post-traumatic stress disorder (PTSD), animal models, microglia, immune system, chemokine

## Abstract

The present study investigates whether predator scent-stress (PSS) shifts the microglia from a quiescent to a chronically activated state and whether morphological alterations in microglial activation differ between individuals displaying resilient vs. vulnerable phenotypes. In addition, we examined the role that GC receptors play during PSS exposure in the impairment of microglial activation and thus in behavioral response. Adult male Sprague Dawley rats were exposed to PSS or sham-PSS for 15 min. Behaviors were assessed with the elevated plus-maze (EPM) and acoustic startle response (ASR) paradigms 7 days later. Localized brain expression of Iba-1 was assessed, visualized, and classified based on their morphology and stereological counted. Hydrocortisone and RU486 were administered systemically 10 min post PSS exposure and behavioral responses were measured on day 7 and hippocampal expression of Ionized calcium-binding adaptor molecule 1 (Iba-1) was subsequently evaluated. Animals whose behavior was extremely disrupted (PTSD-phenotype) selectively displayed excessive expression of Iba-1 with concomitant downregulation in the expression of CX3C chemokine receptor 1 (CX3CR1) in hippocampal structures as compared with rats whose behavior was minimally or partially disrupted. Changes in microglial morphology have also been related only to the PTSD-phenotype group. These data indicate that PSS-induced microglia activation in the hippocampus serves as a critical mechanistic link between the HPA-axis and PSS-induced impairment in behavioral responses.

## 1. Introduction

Post-traumatic stress disorder (PTSD) is an incapacitating chronic syndrome affecting cognitive, emotional, and physiological processing and/or recovery from exposure to a potentially traumatic experience [1]. Most people affected by a potentially traumatic experience will adapt within a period of 1–4 weeks following the trauma, and only a small proportion will develop long-term psychopathology. The development of PTSD is often an evolving process and extends over time through a series of stages starting from relatively contained distress to severe disability. The private, social, and economic burden of PTSD is high and therapeutic approaches are partially effective. Understanding the neurobiological processes, which lead to the development of PTSD symptoms, is critical for the development of effective treatments, and this is accomplished more effectively by using animal models.

Over the past few years, we have developed an animal model of PTSD [2,3,4,5,6,7]. Our model was originally motivated by the actual fact that a clinical diagnosis of PTSD is made only if an individual exhibits a specific number of symptoms of sufficient severity from each of four well-defined symptom clusters over a specific period. The heterogeneity in animal responses might be regarded as a confirmation of the validity of such studies, rather than as a controversy. It stands to reason that a model of diagnostic criteria for psychiatric disorders can be applied to animal responses to strengthen the validity of study data, as long as the criteria for classification are clearly defined, can be reliably reproduced, and yield results that conform to findings in human subjects. In order to model DSM criteria for PTSD, we developed the “Cut-off Behavioral Criteria” model of PTSD-phenotype in rodents. In this translational model, we exposed (adult) rats to a cat urine predator scent stressor (PSS) for 10 min, as previously described by Adamec et al. [8,9] and others [10,11,12,13,14,15,16]. Regarding the conceptual validity of the model itself, “predator scent trauma” is a potentially life-threatening event (criteria A) and may represent a more ‘‘natural’’ condition than other types of stressors, such as electrical tail shocks and restraint on a teleological level that possibly be related to extreme conditions such as torture. Seven days after a single, 10 min PSS exposure, animal behaviors on both the elevated plus maze (EPM) and the acoustic startle response (ASR) test were evaluated and served as tools for classifying individuals as exhibiting either an “extreme behavioral response” (EBR), a “minimal behavioral response” (MBR), or—midway between EBR and MBR—a “partial behavioral response” (PBR). The creation of clearly defined groups of animals enables the statistical analysis of the “prevalence rate” of each specific pattern of behavioral response. The proportion of the entire exposed population fulfilling the criteria for extreme responses (EBR) (25%) is compatible with epidemiological data for PTSD amongst trauma-exposed human populations [17]; these data report that between 15 and 35% fulfill the criteria for PTSD and that approximately 20–30% display partial or sub-symptomatic clinical pictures [18]. This compatibility further supports the idea of criterion-based behavioral classification in terms of face validity. Our animal model has demonstrated high face, construct, and predictive validity [2,4,5,19,20,21,22,23,24,25,26,27,28,29,30,31,32,33,34].

To explore the molecular signature underlying these individual differences, we performed genome-wide expression profiling in blood, amygdala, and hippocampus [33]. An upstream regulator analysis (URA) using the differentially expressed gene (DEG) signatures computationally predicted activated/de-activated upstream transcription factors (TFs). Nine regulators were conserved in both blood and brain tissues, including the glucocorticoid receptor (GR) and also the IκBα (encoded by NFKBIA), an inhibitor of nuclear factor-κB (NF-κB). The GR signaling pathway was identified as one of the most convergent pathways associated with extreme differences in the behavioral response to PSS exposure. In addition, the NF-κB pathway mediates inflammation in the brain orchestrating transcriptional cascades that lead to changes in neuronal structure and function. Consistent with this idea, increased hippocampal NF-κB signaling has been associated with vulnerability to PSS [22] and up-regulated in PTSD [35], and could be prevented by glucocorticoids (GCs) or other more specific inhibitors [22]. We extended our URA of the above-mentioned amygdala, hippocampus, and blood DEG signatures associated with individual differences to predict the 38 cytokines and 22 growth factors that regulate these genes. A brain–blood overlap revealed the blood regulators with functional relevance to brain phenotypes; 78.6% of brain cytokines and 47.6% of brain growth factors were recapitulated in blood, whereas eight cytokines (IFN-γ, interleukin-1β (IL-1β), IL-3, IL-5, IL-27, prolactin, tumor necrosis factor α (TNF-α), tumor necrosis factor ligand superfamily member 11) and five growth factors (angiotensinogen, epidermal growth factor, fibroblast growth factor 2, nerve growth factor, TGF-β1) were convergent across tissues.

Given the close relationship between the immune and neuroendocrine systems, it is reasonable to hypothesize that the immune system plays a central role in PTSD pathophysiology. A first meta-analysis indicated that IFN-γ, IL-1β, IL-6, and TNF-α are the most consistently elevated pro-inflammatory cytokines in the blood of patients with PTSD compared with healthy controls [36]. Moreover, unbiased genome-wide analyses found altered DNA methylation and RNA expression in multiple genes related to immune function in subjects with PTSD [35,37,38,39]. Finally, in soldiers, higher GC-dependent cytokine production and T-cell proliferation before deployment are associated with increased PTSD symptoms after combat [40]. These findings suggest that immune factors might not only be markers for symptom state, but also contribute to pre-existing risks for PTSD upon trauma exposure. However, neither the mechanistic origin(s) nor the functional relevance of PTSD-associated inflammation are fully understood.

Stress directly influences microglial cell morphology and function [41,42,43,44,45,46,47,48,49,50,51,52]. Several studies show an association between hyperactive microglial and behavioral outcomes that are proxies for depressive and anxiety-like symptoms in animal models. Studies by Wohleb [53,54] using the social defeat model showed that anxiety-like behavior is positively associated with microglial activity. Couch et al. [55], using a model that combined social defeat, restraint, and tail suspension, observed a pro-inflammatory profile and heightened microglial activity associated with the development of stress-induced anhedonia in mice susceptible to depression following exposure to stress. These changes in the microglial profile were not present in “resilient” animals that did not develop depressive symptoms following the stress protocol [56]. There have been few studies on whether stress alone, without an additional immune challenge, might alter microglial activation. Nair et al. [43] demonstrated that restraint stress activates microglia and induces microglia proliferation; however, very few studies have reported on stress-induced sensitization of microglia pro-inflammatory responses in PTSD. Recently, Sun et al. [57] reported that stress-induced microglia activation in the hippocampus may serve as a critical mechanistic link in the comorbid relationship between PTSD and chronic pain. Together, these studies provide support for the hypothesis that immune dysregulation and microglial activation are involved in the neurobiology of stress-related disorders. However, there is no research to date investigating the role of microglia in the development of the PTSD phenotype.

The aim of the current study was to determine the role of microglia in the development of the PTSD phenotype in rats exposed to PSS and to elucidate the mechanisms underlying stress-induced inflammation that contribute to the PTSD phenotype.

## 2. Results

### 2.1. Effects of Predator Scent-Stress (PSS) Exposure on Microglia

**Figure 1:**
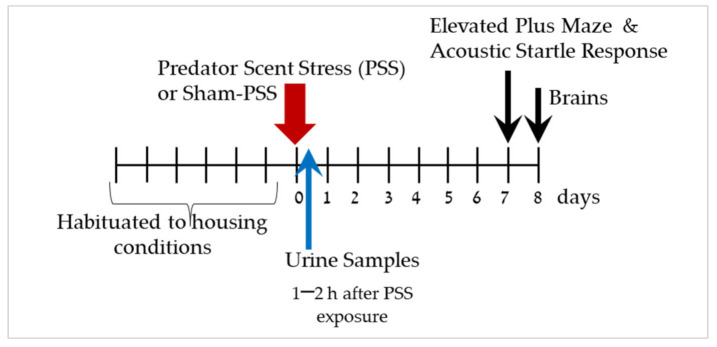
Study design—The effect of predator scent-stress (PSS) on corticosterone levels and behavioral responses. After habituation period of seven days, animals were exposure to PSS or sham-PSS for 15 min. Urine samples were collected 1–2 h after PSS exposure for corticosterone levels. Behaviors were assessed on day 7 using the elevated plus maze (EPM) and the acoustic startle response (ASR) assay. On day 8, rats were sacrificed, and their brains were collected for immunoreactivity analyses of Iba-1 and CRXCR1 in the hippocampus subregions.

The experimental design used here is schematically depicted in Figure 1.

#### Effects of Predator Scent-Stress Exposure on Behavioral Responses at Day 7 Post Exposure

**Figure 2:**
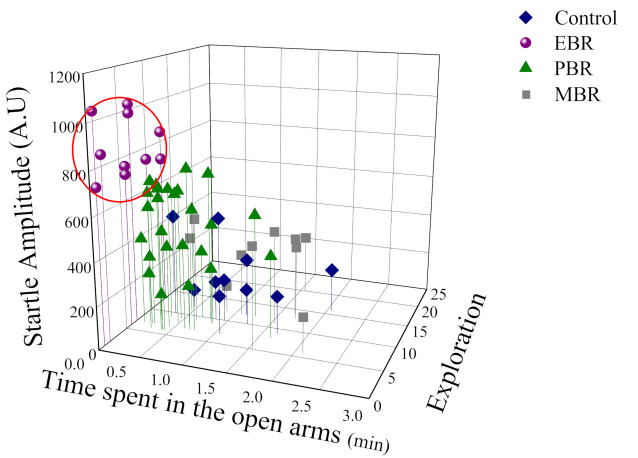
The effect of the predator scent stress (PSS) paradigm on overall anxiety-like behavior and startle response: Three-dimensional parameters: The X-axis represents time spent in the open arms (min), the Y-axis represents acoustic startle amplitude, and the Z-axis—total exploration on the maze. The stress response was not homogeneous, and several subgroups were identifiable in the population. Circles (purple) represent the exposed group that exhibited a significant degree of anxiety-like and avoidant behaviors on the elevated plus-maze and a pattern of exaggerated startle responses with significantly reduced habituation 7 days after exposure (PTSD-like phenotype). Triangles (green) represent the exposed group that exhibited partial behavioral response patterns. Squares (gray) represent the exposed group that exhibited minimal behavioral disrupted. Diamonds (blue) represent the un-exposed control group.

As shown in Figure 2, a broad range of behavioral response variation was observed within the PSS-exposed group, and several subgroups were identifiable. Based on this study phase results, the animals were subdivided into groups reflecting the magnitude of response according to the CBC’s, focusing selectively on EBR, PBR, and MBR.

In the exposed group 22.22% (10/45) fulfilled criteria for EBR, 55.55% (25/45) for PBR and 22.22% for MBR (1/450). Based on this study phase results, the animals were subdivided into groups reflecting the magnitude of response according to the CBCs, focusing selectively on EBR, PBR, and MBR.

### 2.2. Effects of Predator Scent-Stress Exposure on Corticosterone Levels at 1–2 h after Exposure

**Figure 3:**
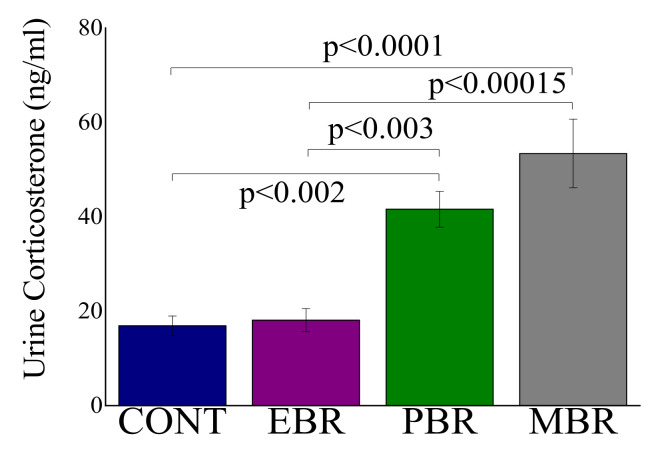
Urine levels of corticosterone at 1–2 h after PSS-exposure or sham-PSS: All data represent the group means ± S.E.M.

Significant differences in urine corticosterone levels were demonstrated between the subgroups of PSS exposed animals (F(3, 51) = 12.5, *p* < 0.0001). Bonferroni post hoc tests indicated that the EBR group exhibited a significantly blunted corticosterone response to PSS compared with MBR (*p* < 0.00015) and PBR (*p* < 0.003) groups (Figure 3).

### 2.3. Iba-1 Positive Cell Counts in Hippocampal Subregions at Day 8 Post PSS-Exposure

**Figure 4:**
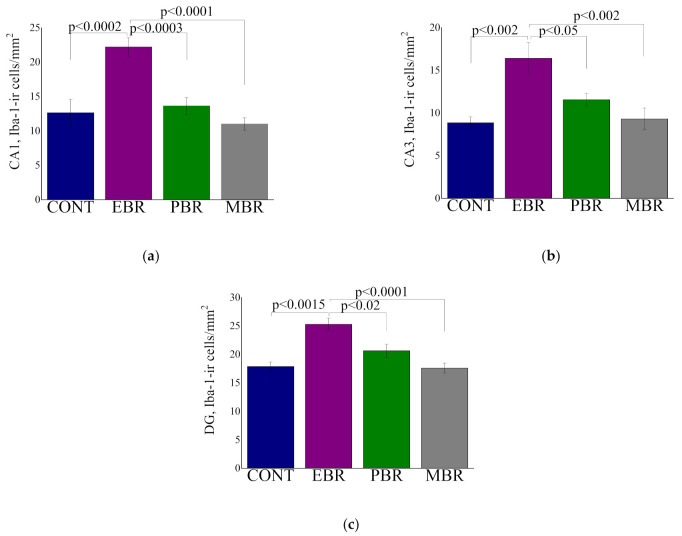
The quantitative morphometric analysis of Iba-1 immunoreactivity (ir) in cells of the hippocampal subregions CA1 (**a**), CA3 (**b**), and DG (**c**) in unexposed controls (CONT) in animals with an extreme behavioral response (EBR) or partial response (PBR) to the PSS exposure, and in animals whose behavior was minimally affected by the stressor (MBR). All data represent the group means ± S.E.M.

Significant differences were noted in the CA1 (Figure 4a), CA3 (Figure 4a,b), and DG (Figure 4c) subregions in the expression of Iba-1-ir cells levels between groups (F(3, 35) = 14.1, *p* < 0.0001, F(3, 35) = 7.4, *p* < 0.0006 and F(3, 35) = 11.9, *p* < 0.00001, respectively) (Figure 4). Bonferroni post hoc revealed that the EBR group had significantly higher cell density in the hippocampal subregions CA1, CA3 and DG compared with that in control (*p* < 0.0002, *p* < 0.002 and *p* < 0.0002, respectively), PBR (*p* < 0.0003, *p* < 0.05 and *p* < 0.015, respectively), and MBR (*p* < 0.0001, *p* < 0.002 and *p* < 0.0001, respectively) rats.

### 2.4. Morphological Analysis of Iba-1 in CA1, CA3 and DG Hippocampal Subregions at Day 8 Post PSS-Exposure

In the CA1 hippocampal subregion, significant differences were noted in the number of microglia branches between groups (F(3, 35) = 3.56, *p* < 0.025) (Table 1). Bonferroni post hoc revealed that the number of branches in the control group was significantly higher compared with the EBR group (*p* < 0.0025). Significant differences were noted in the length of the microglia branches between groups (F(3, 35) = 4.9, *p* < 0.0065). Bonferroni post hoc revealed that the branches in the control group were significantly longer compared with EBR (*p* < 0.0045) and PBR (*p* < 0.05) groups. Significant differences in the soma size between the groups (F(3, 35) = 4.13, *p* < 0.015). Bonferroni post hoc revealed that the soma size in the EBR group was larger compared with PBR (*p* < 0.025), and MBR (*p* < 0.05) groups.

In the CA3 hippocampal subregion, significant differences were noted in the number of microglia branches between groups (F(3, 35) = 45.3, *p* < 0.0001). Bonferroni post hoc revealed that the number of branches in the EBR group was significantly lower compared with control (*p* < 0.0001), PBR (*p* < 0.005), and MBR (*p* < 0.0001) groups. The number of branches in the PBR group was significantly lower compared with MBR (*p* < 0.0001) group. The number of branches in the MBR group was significantly higher than the control (*p* < 0.0002) group. Significant differences were noted in the length of the microglia branches between groups (F(3, 351) = 48.7, *p* < 0.0001). Bonferroni post hoc revealed that the EBR group branches were significantly shorter than to control (*p* < 0.0001), PBR (*p* < 0.0001), and MBR (*p* < 0.0001) groups. In addition, the branches in the PBR group were significantly shorter compared with the MBR (*p* < 0.0001) group. The MBR group branches were significantly longer compared with the control (*p* < 0.0035) group. No significant differences in the soma size between the groups

In the DG hippocampal subregion, significant differences in the number of microglia branches between the groups (F(3, 35) = 7.9, *p* < 0.0004). Bonferroni post hoc revealed that the number of branches in the EBR group was significantly lower compared with control (*p* < 0.0001), and MBR (*p* < 0.015) groups. Significant differences in the microglia length between the groups (F(3, 35) = 7.9, *p* < 0.0004). The brunches in the EBR group were significantly shorter compared with PBR groups (*p* < 0.0045). Significant differences in the soma size between the groups (F(3, 35) = 4.8, *p* < 0.0065). Bonferroni post hoc revealed that the soma size in the EBR group was larger compared with the PBR (*p* < 0.0045) group.

#### Sholl Analysis of Iba-1 in CA1, CA3 and DG Hippocampal Subregions at Day 8 Post PSS-Exposure

The number of Sholl intersections, points where microglia cross the virtual Sholl shells, is a parameter that reflects the complexity of the branches tree.

**Figure 5:**
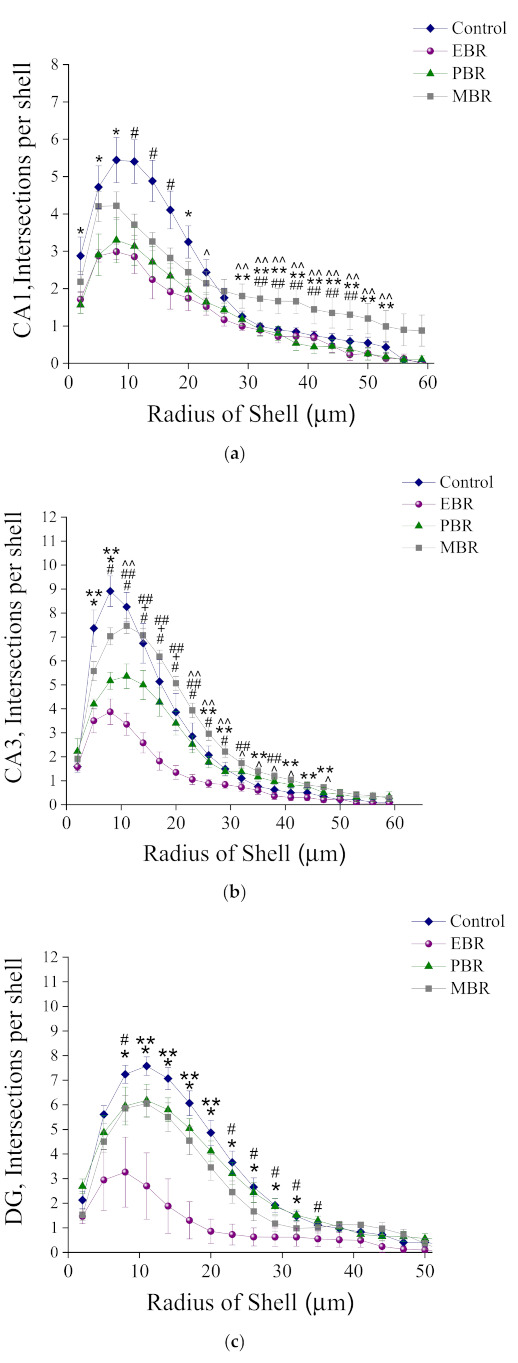
Sholl-analysis for intersections per 3 mm radial unit distance from sham-PSS, EBR, PBR, and MBR animals. Results displayed as mean ± S.E.M. (**a**) CA1 hippocampal subregion at day 8 post PSS-exposure: * Control ≠ EBR and PBR, *p* < 0.04. # Control ≠ EBR, PBR and MBR, *p* < 0.03. ^ Control ≠ EBR, *p* = 0.04. ** EBR ≠ MBR, *p* < 0.04. ^^ PBR ≠ MBR, *p* < 0.03. ## Control ≠ MBR, *p* < 0.04. (**b**) CA3 hippocampal subregion at day 8 post PSS-exposure: * Control ≠ EBR and PBR, *p* < 0.04. # Control ≠ EBR, PBR and MBR, *p* < 0.03. ^ Control ≠ EBR, *p* = 0.04. ** EBR ≠ MBR, *p* < 0.04. ^^ PBR ≠ MBR, *p* < 0.03. ## Control ≠ MBR, *p* < 0.04. + Control ≠ PBR, p < 0.05. (**c**) DG hippocampal subregion at day 8 post PSS-exposure: * Control ≠ EBR and PBR, *p* < 0.04. # Control ≠ EBR, PBR and MBR, *p* < 0.03. ^ Control ≠ EBR, *p* = 0.04. ** EBR ≠ MBR, *p* < 0.04. ^^ PBR ≠ MBR, *p* < 0.03. ## Control ≠ MBR, *p* < 0.04.

In the CA1 hippocampal subregion: In response to PSS, microglia from EBR, PBR groups had significantly fewer dendritic intersections with each sphere at Sholl radii of 2–23 μm than did microglia from sham-PSS controls. In addition, microglia from EBR, PBR and Control groups had significantly fewer dendritic intersections with each sphere at Sholl radii of 29–53 μm than did microglia from MBR group 2-way ANOVA: groups: F(3, 880) = 38.36, *p* < 0.0001, Radius: F(19, 880) = 50.44, *p* < 0.0001, and Group-radius interaction: (F(57, 880) = 2.72, *p* < 0.0001; (Figure 5a)). No differences were detected at any radii between EBR, PBR, and MBR animals.

In the CA3 hippocampal subregion: In response to PSS, microglia from the EBR group had significantly fewer dendritic intersections with each sphere at Sholl radii of 5–47 mm than did microglia from sham-PSS controls and PBR and MBR groups. Most of the radii were noted a pattern of significantly fewer intersections in the Control group compared with the MBR group and fewer intersections in the PBR group compared with the MBR group 2-way ANOVA: groups: (F(3, 880) = 138.91, *p* < 0.001, Radius: F(19, 880) = 159.37, *p* < 0.001, and Group-radius interaction: F(57, 880) = 8.85, *p* < 0.001; Figure 5b).

In the DG hippocampal subregion: In response to PSS, microglia from EBR had significantly fewer dendritic intersections with each sphere at Sholl radii of 8–35 μm than did microglia from sham-PSS controls, PBR and MBR groups 2-way ANOVA: Groups: F(3, 880) = 55.86, *p* < 0.0001, Radius: F(19, 880) = 79.03, *p* < 0.001, and Group-radius interaction: F(57, 880 = 3.0, *p* < 0.0001; Figure 5c).

### 2.5. Correlation Analysis between Corticosterone Levels at the Initial PSS Response and Microglia Measures at Day 8 Post Exposure

**Figure 6:**
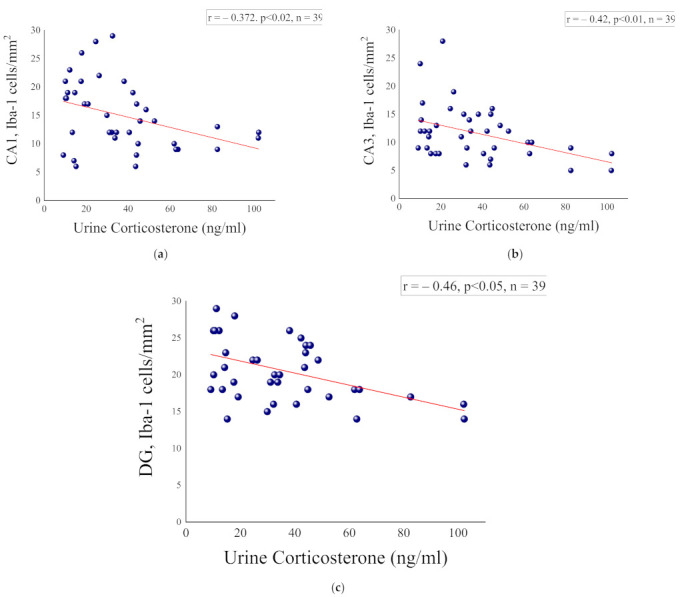
Correlation analysis between hippocampal microglia measures and urine corticosterone levels. (**a**) CA1 hippocampal subregion and urine corticosterone, (**b**) CA3 hippocampal subregion and urine corticosterone and (**c**) DG hippocampal subregion and urine corticosterone.

Figure 6a–c contains the results of a correlation analysis involving the microglia cells (8 days after PSS exposure) and urine corticosterone levels (collected 1–2 h after PSS exposure). Pearson’s correlation analysis revealed that the CA1 subregion iba-1-ir cell number was significantly correlated with the urine corticosterone levels (r = −0.43, *p* < 0.007).

### 2.6. CX3CR1-ir Cells in Hippocampal Subregion at Day 8 Post PSS-Exposure

**Figure 7:**
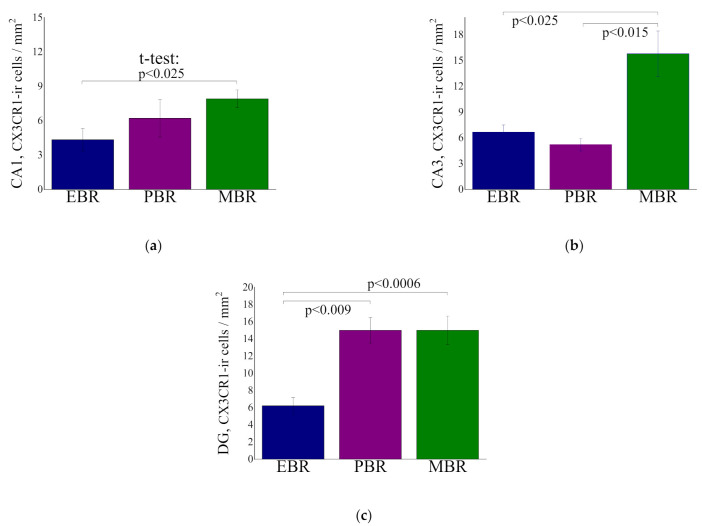
Quantitative analysis of CX3CR1-ir cells in the CA1 (**a**), CA3 (**b**) and DG (**c**) hippocampal subregions at day 8 post PSS-exposure in animals with an extreme behavioral response (EBR) or partial response (PBR) to the PSS exposure, and in animals whose behavior was minimally affected by the stressor (MBR). All data represent the group means ± S.E.M.

CA1 hippocampal subregion: One-way ANOVA analysis revealed no significant differences in the number of CX3CR1-ir cells between the groups (F(2, 18) = 3.2, *p* = 0.066), whereas a trend towards a lower number of CXCR1 in the EBR group compared with the MBR group was noted (*p* < 0.025) (Figure 7a).

CA3 hippocampal subregion: One-way ANOVA analysis revealed significant differences in the number of CX3CR1-ir cells between the groups (F(2, 17) = 7.5, *p* < 0.005). Bonferroni post hoc revealed that the number of CX3CR1 in the EBR group was significantly lower compared with MBR (*p* < 0.0025) group. In addition, the number of CX3CR1 in the PBR group was significantly lower compared with MBR (*p* < 0.015) group (Figure 7b).

DG hippocampal subregion: One-way ANOVA analysis revealed significant differences in the number of CX3CR1-ir cells between the groups (F(2, 26) = 10.5, *p* < 0.00045). Bonferroni post hoc revealed that the number of CX3CR1 in the EBR group was significantly lower compared with PBR (*p* > 0.009) and MBR (*p* < 0.0006) group (Figure 7c).

### 2.7. Effects of High-Dose Hydrocortisone, RU486 (GR Receptor Antagonist) or Saline Immediately Post-Exposure on Behavioral Stress Responses at Day 7 Post-Exposure

**Figure 8:**
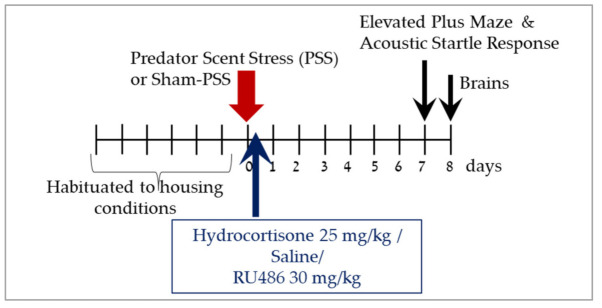
Study design—Effects of high-dose hydrocortisone, RU486 (GC receptor antagonist-mifepristone) or saline immediately post-exposure on behavioral stress responses at day 7 post-exposure. After habituation period of seven days, animals were exposure to PSS or sham-PSS for 15 min. Animals were exposed to predator scent stress (PSS), treated for 1 day with hydrocortisone 25 mg/kg, saline or RU486 30 mg/kg and assessed behaviorally with the elevated plus maze (EPM) and acoustic startle response (ASR) tests at day 7. On day 8, rats were sacrificed, and their brains were collected for immunoreactivity analyses of Iba-1 in the hippocampus subregions.

The experimental design used here is schematically depicted in Figure 8.

### 2.8. Effects of High-Dose Hydrocortisone, Saline or RU486 Early Post-Exposure on Behavioral Stress Responses at Day 7 Post-Exposure

**Figure 9:**
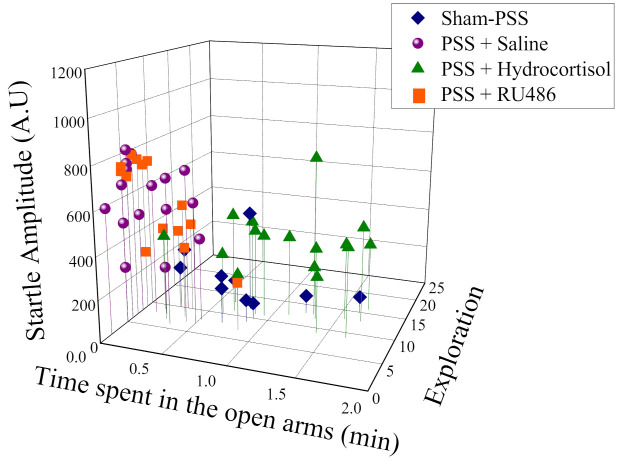
High-dose hydrocortisone, saline or RU486 early post-exposure on behavioral stress responses at day 7 post-exposure: Three-dimensional parameters: The X-axis represents time spent in the open arms (min), the Y-axis represents acoustic startle amplitude (A.U), and the Z-axis—total exploration on the maze. Circles (purple) represent the exposed group that treated with Saline (*n* = 16), which exhibited a significant degree of anxiety-like and avoidant behaviors on the elevated plus-maze and a pattern of exaggerated startle responses with significantly reduced habituation 7 days after exposure (PTSD-like phenotype). Triangles (green) represent the exposed group that treated with Hydrocortisone 25 mg/kg (*n* = 16), which exhibited minimal or partial behavioral response patterns. Diamonds (blue) represent the sham-PSS-exposed control group (*n* = 10). Squares (orange) represent the exposed group that treated with RU486 30 mg/kg (*n* = 14), which exhibited extreme or partial behavioral response patterns.

As shown in Figure 9, comparison between the groups (not comparing individuals within each groups) revealed that exposed groups treated with saline or RU486 exhibited a significant decrease in overall time spent in the open arms (F(3, 52) = 32.4, *p* < 0.0001), a significant decrease in the number of entries to open arms (F(3, 52) = 18.2, *p* < 0.0001), and a significantly increased mean startle amplitude (F(3, 52) = 15.6, *p* < 0.0001) compared with sham-PSS controls (PSS-Saline: *p* < 0.0001, *p* < 0.0002, and *p* < 0.0001, respectively and PSS-RU486: *p* < 0.0002, *p* < 0.0001 and *p* < 0.0001, respectively) and to exposed rats treated with hydrocortisone (PSS-Saline: *p* < 0.0001, *p* < 0.0001 and *p* < 0.008, respectively and PSS-RU486: *p* < 0.0001, *p* < 0.0001 and *p* < 0.04, respectively).

**Relative prevalence rates according to CBC:** Significant differences in the prevalence rates of individual rats displaying extreme, partial, or minimal responses were found among the groups (Pearson χ^2^ = 14.7, df = 6, *p* < 0.025).

There were significant differences in the prevalence rates of individuals displaying EBR among groups (Pearson χ^2^ =12.2, df = 3, *p* < 0.05). The prevalence of EBR rats among PSS-exposed rats injected with saline was 25.0% of the total rats and differed significantly from that among PSS-exposed rats treated with hydrocortisone, in which there were no EBR rats (χ^2^ = 4.57, *p* < 0.035). PTSD-like EBRs were significantly more prevalent in rats exposed to PSS and treated with RU486 (6/14 rats, 42.85%) than in rats exposed to PSS and treated with hydrocortisone (0/16 rats; χ^2^ = 8.57, *p* < 0.0035) or to sham-PSS group (χ^2^ = 5.71, *p* < 0.02). There were significant differences in the prevalence rates of individuals displaying MBR among groups (Pearson χ^2^ =8.04, df = 3, *p* < 0.05).

The prevalence rates of MBR among the PSS-exposed rats treated with hydrocortisone was 25% (4/16) of the total rats and differed from that among PSS-exposed animals treated with saline (χ^2^ = 4.57, *p* < 0.0325), or treated with RU485 (χ^2^ = 4.04, *p* < 0.045), in which there were no MBR rats. There were no significant differences in the prevalence of PBR among groups. Taken together, these analyses indicate that treating rats with hydrocortisone following exposure to a traumatic event induces a significant shift towards less extreme behavioral disruption, suggesting that the treatment confers some degree of protection against the trauma-related sequelae.

### 2.9. Effects of High-Dose Hydrocortisone, Saline or RU486 Early Post-Exposure on Iba-1 Positive Cell Counts in Hippocampus

Significant differences were noted in the number of microglia branches in hippocampal subregions CA1 (F(3, 52) = 14.4, *p* < 0.0001), CA3 (F(3, 52) = 13.8, *p* < 0.0001) and DG (F(3, 52) = 8.0, *p* < 0.0003) between groups, as shown in Table 2. Bonferroni post hoc test revealed that the number of branches in the exposed group treated with hydrocortisone as well as in the control group (sham-PSS) was significantly higher compared with exposed group treated with saline (CA1: *p* < 0.0001 and *p* < 0.002, respectively; CA3: *p* < 0.0001 and *p* < 0.0002, respectively; DG: *p* < 0.0035 and *p* < 0.006, respectively) or exposed rats treated with RU486 (CA1: *p* < 0.0001 and *p* < 0.009, respectively; CA3: *p* < 0.0008 and *p* < 0.0035, respectively; DG: *p* < 0.02 and *p* < 0.0035, respectively).

Significant differences were noted in the length of the microglia branches in hippocampal subregions CA1 (F(3, 52) = 21.0, *p* < 0.0001), CA3 (F(3, 52) = 24.8, *p* < 0.0001) and DG (F(3, 52) = 11.4, *p* < 0.0001) between groups. Bonferroni post hoc revealed that the branches in the exposed group treated with hydrocortisone and in the sham-PSS control group were significantly longer compared with exposed group treated with saline (CA1: *p* < 0.0001 for both groups; CA3: *p* < 0.002 for both groups; DG: *p* < 0.0035 and *p* < 0.0008, respectively) or exposed rats treated with RU486 (CA1: *p* < 0.0001 for both groups; CA3: *p* < 0.0001 and *p* < 0.003, respectively; DG: *p* < 0.0008 and *p* < 0.0002, respectively). No significant differences in the soma size between the groups.

Significant differences in the soma size in hippocampal subregions CA1 (F(3, 52) = 3.3, *p* < 0.03), CA3 (F(3, 52) = 7.5, *p* < 0.0003) and DG (F(3, 52) = 3.0, *p* < 0.04) between groups.

Bonferroni post hoc revealed that in the CA3 and DG subregions the soma size in the exposed group treated with RU496 was larger compared with exposed group treated with hydrocortisone (*p* < 0.0015 and *p* < 0.05, respectively). In addition, in the CA1 subregion the soma size in the exposed group treated with RU496 was larger compared with sham-PSS controls (*p* < 0.05).

### 2.10. Effects of High-Dose Hydrocortisone, Saline or RU486 Immediately Post-Exposure on Iba-1 Morphology

**Figure 10:**
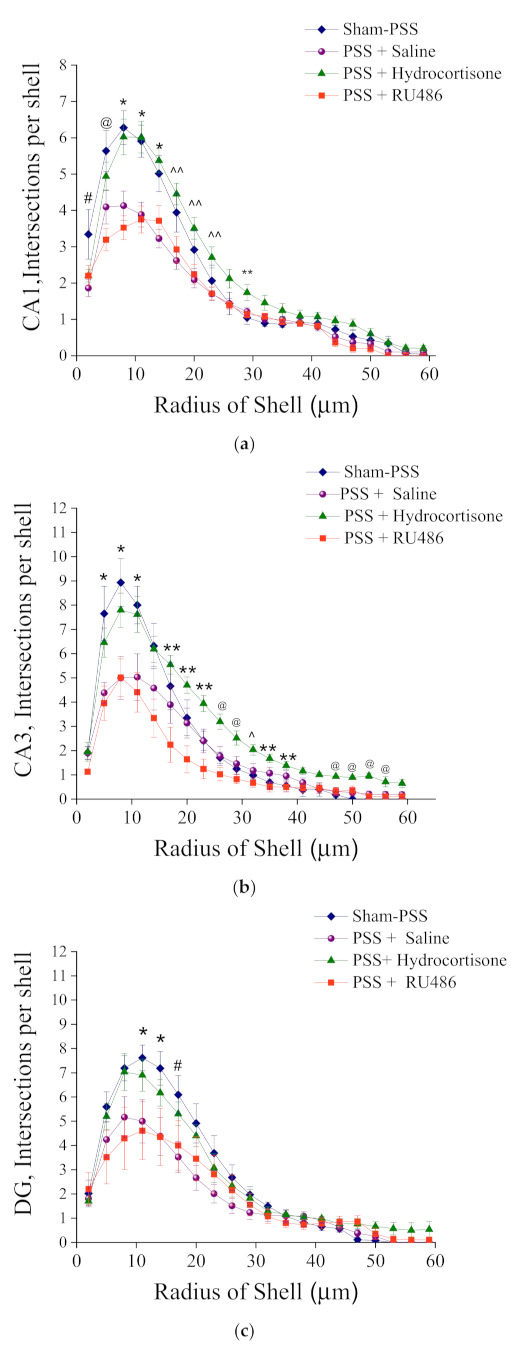
Sholl-analysis for intersections per 3 mm radial unit distance from sham-PSS, PSS +Saline, PSS + Hydrocortisone 25 mg/kg and PSS + RU486 30 mg/kg groups. Results displayed as mean ± S.E.M. (**a**) CA1 hippocampal subregion at day 8 post PSS-exposure: # Sham-PSS ≠ PSS-Saline-*p* < 0.025. * Sham-PSS, PSS-Hydrocortisone ≠ PSS-Saline, PSS-RU486-*p* < 0.015. ^^ PSS-Hydrocortisone ≠ PSS-Saline, PSS-RU486-*p* = 0.03. ** Sham-PSS ≠ PSS-Hydrocortisone-*p* < 0.04. @ PSS-RU486 ≠ Sham-PSS, PSS-Hydrocortisone *p* < 0.03 (**b**) CA3 hippocampal subregion at day 8 post PSS-exposure: * Sham-PSS ≠ PSS-Saline, PSS-RU486-*p* < 0.05. ** PSS-Hydrocortisone ≠ PSS-RU486-*p* < 0.05. @ PSS-Hydrocortisone ≠, Sham-PSS, PSS-Saline, PSS-RU486-*p* < 0.045. ^ PSS-Hydrocortisone ≠ PSS-Saline, PSS-RU486-*p* = 0.05. (**c**) DG hippocampal subregion at day 8 post PSS-exposure: * Sham-PSS ≠ PSS-Saline, PSS-RU486-*p* < 0.04. # Sham-PSS ≠ PSS-Saline-*p* < 0.02.

Significant differences were found in the number of intersections between groups (CA1: Groups: F(3, 1040) = 61.2, *p* < 0.00001; Radius: F(19, 1040) = 94.4, *p* < 0.0001; Groups x Radius: F(57, 1040) = 3.5, *p* < 0.00001; CA3: Groups: F(3, 1040) = 43.2, *p* < 0.00001; Radius: F(19, 1040) = 88.2, *p* < 0.0001; Groups x Radius: F(57, 1040) = 2.4, *p* < 0.00001; and DG: Groups: F(3, 1040) = 16.0, *p* < 0.00001; Radius: F(19, 1040) = 69.1, *p* < 0.0001; Groups x Radius: F(57, 1040) = 1.4, *p* < 0.025). Sholl analysis for intersections per 3 mm radial unit distance showed that microglia from exposed group treated with saline or RU486 had significantly fewer branches’ intersections within each sphere at Sholl radii of 5–30 mm than did branches from sham-PSS controls, or exposed group treated with hydrocortisone (Figure 10a,b).

In the DG region, Bonferroni post hoc revealed that microglia from he exposed groups that treated with saline or RU486 had significantly fewer dendritic intersections with each sphere at Sholl radii of 11–17 μm than did microglia from sham-PSS controls, or exposed group treated with hydrocortisone (Figure 10c).

## 3. Discussion

To elucidate the role of microglial involvement in the underpinnings of stress response processes, we evaluated whether PSS induces long-term microglial proliferation and what factors could lead to a long-term “reactive microglial” phenotype.

### 3.1. Microglia Proliferation

In our study, we examined changes in microglial number (Iba-1) in hippocampal subregions (CA1, CA3, DG) in response to psychological stressor, in Sprague Dawley male rats. We found that the development of an extreme long-term behavioral response pattern (PTSD-phenotype) at 8 days post PSS exposure was associated with a distinct pattern of long-term regionally distinct changes in the expression of Iba-1 in the hippocampal sub-regions. Iba-1 positive cell counts revealed that the PTSD-phenotype group had a significantly higher cell density in the hippocampal subregions than in PBR, and MBR rats and compared with sham-PSS controls. This suggests that PSS was creating a pro-inflammatory environment within the hippocampus in PTSD phenotype rats.

These results are in accordance with previous studies that have reported the crucial role of pro-inflammatory microglia activation in mediating neuropsychiatric disorders, including PTSD. Countless studies reported that chronic stress (i.e., inescapable shock or/and water immersion or/and restraint) induced a microglia proliferation and a significant increase in the density of microglia immunolabeling in the brain [43,46,48,49,58]. In addition, studies indicate that chronic stress with an additional immune challenge (such as lipopolysaccharides) alters microglial activation and increases their density [44,45]. This is the first report of those findings in a PTSD animal model where one-time exposure occurred; however, Nair and Bonneau [43], indicated that four-time exposure (per day) to stress is required to increase the microglia density, and on day 5–6 decreasing in density occur. Those inconsistent results with this study may drive by (a) different stress models applied-chronic stress v PSS, and/or (b) microglia cell death happening after 5 times of exposure per day.

One may speculate that the increased numbers of Iba-1-ir cells could result from proliferation since microglia can proliferate rapidly after being activated [43,59]. This may have harmful effects on the brain, especially if these microglia become pathogenic [60]. This is interesting because recent studies show that microglia modulate neuroplasticity [61,62], thereby raising the likelihood that stress-induced microglial activation contributes to synaptic deficits underlying the PTSD phenotype [23].

### 3.2. Microglia Morphology

In the next step, we examined the morphology of microglia in hippocampal subregions of the brain, using image analysis of highly magnified cell drawings. We found changes in microglial morphology have also been related only to the PTSD-phenotype group. The microglial cells in the hippocampus subregions showed decreased branching and increased nuclear area. Taken together, we found that PSS exposure shifts the state of microglia from quiescent to activated only in the PTSD-phenotype group. These microglia exhibited long-term regionally distinct changes in expression and proliferation in the hippocampus in response to PSS exposure.

Several studies show an association between hyperactive microglial and behavioral outcomes similar to PTSD [52,53,54,57]. Studies using restraint models [43,63,64] show an increase in the number of microglia cells, microglial branching, the number of processes, and process length, as the microglia exhibit a ramified morphology. This is one stage before the “pathogenic” stage of microglia. Our results suggest microglia stays activated in PTSD phenotype individuals until it became pathogenic. Studies by Diz-Chaves [44,45] using prenatal stress and induced lipopolysaccharides revealed that treated mice had larger somas and thicker processes compared with untreated. Even though there is a great deal of evidence of microglia activation, this is the first study to investigate the role of stress-induced inflammation that causes microglia activation and the ramifications on the development of PTSD.

Initial studies in rodent models showed that stress exposure increased pro-inflammatory cytokine levels in the brain. Given the above, PSS exposure increased pro-inflammatory cytokines (such as IL-1α, TNF and IL-6) in the hippocampus, hypothalamus and frontal cortex, with the increase still present 8 days after stressor termination [65]. In the brain, these cytokines activate microglial cells and induce the production of more cytokines. The process results in a positive feedback loop, which can become self-sustaining and cause systemic organ dysfunction [66]. Furthermore, the induction and penetration into the brain of pro-inflammatory cytokines during stress reactions may by itself induce anxiogenic effects, creating a self-propagating loop between (psychological) stress and immune activation. Once in the brain, cytokines and microglia affect neuronal synaptic plasticity by modifying cell signaling and gene expression [67].

### 3.3. CX3XR1 Levels

However, the manner in which microglia are activated may be pivotal in determining how they impact the outcome of PSS exposure. It is well known that under normal/healthy conditions, microglia are regulated by soluble factors released by neurons, including CX3CL1, colony-stimulating factor 1, CD200, etc. [68,69]. Some of these regulatory factors may act on the CNS-resident microglia (or meningeal macrophages), shifting their phenotype to a pro-inflammatory state, thereby indirectly affecting neuronal networks and resulting in severe behavioral disruption (i.e., EBR). Therefore, we tested the possibility that PSS may weaken inhibitory control over microglia, thereby permitting these cells to shift from a surveillant state to a primed state of activation. We hypothesized that impaired CX3CR1 function would be associated with microglial priming/activation in specific brain regions, impacting behaviors relevant to PTSD. Therefore, we examined changes in CRXCR1 levels in hippocampal subregions (CA1, CA3, and DG) in this animal model.

We found that the development of an extreme long-term behavioral response pattern at 8 days post PSS exposure was associated with a distinct pattern of long-term regionally distinct changes in the expression of CRXCR1 in the hippocampal subregions. CRXCR1 positive cell counts revealed that the PTSD-phenotype group had a significantly lower cell density in the CA3 and DG hippocampal subregions than in PBR, and MBR rats. In response to PSS, disruption of CRXCR1 is sufficient to cause dysregulation of microglial cell homeostasis and hence of neuronal networks, resulting in severe behavioral disruption.

These results align with existing evidence in mouse models. CX3CR1 signaling acts as a protector against microglial neurotoxicity, and CX3CR1 deficiency dysregulates microglial responses, resulting in neurotoxicity [70]. These results are shown in a mouse model of Alzheimer’s disease [59,71], in ischemic mice brain [72,73], and a rat model of Parkinson’s disease [74].

It is interesting to note that such a microglia shift (morphological transformations and altered functionality) also occurs during aging [75] and may potentially contribute to decreased cognitive functions. For example, Cx3cr1 deficiency in mice led to a greater microglia activation, diminished LTP (synaptic plasticity), and deficits in hippocampal-dependent cognitive function [76]. Cognitive and learning deficits resulting from microglia ablation highlight the importance of properly functioning microglia for higher order processes in the adult brain [77,78].

However, the signals leading to priming/activating of microglia in response to PSS remain unclear. Studies by others, as well as our results, indicate that stress, through GC action, may enhance central nervous system (CNS) inflammation [43,52]. Therefore, we hypothesize that PSS-induced elevation of GCs initiates a cascade of events in the CNS, leading to microglial proliferation: PSS exposure results in immediate HPA axis activation and elevations in endogenous GCs. GCs, via direct or indirect pathways, may act as a neuroendocrine danger signal and initiate a process that sensitizes microglia [52].

### 3.4. Urine Corticosterone Levels Immediately after PSS Exposure

In the next step, we determined if urine corticosterone levels during PSS exposure correlate with microglial activation and with distinct behavioral response patterns, i.e., the EBR, PBR, and MBR phenotype. In response to PSS, we found significant differences in corticosterone profile among groups. Extreme disruption of behavior (PTSD-phenotype) on day 8 post-exposure was characterized by blunting of corticosterone response to the stressor, both of which were not present in minimal or partial behavioral responders (MBR or PBR). We conducted regression analyses to gain a further understanding of the relationship between corticosterone levels (during PSS) and Iba-1 levels (8 days post-PSS) in the hippocampus irrespective of the CBC classification. Pearson’s correlation analysis revealed that corticosterone levels were significantly and negatively correlated with the CA1 hippocampal Iba-1 levels. These results align with the literature, as lower baseline cortisol levels after stress exposure have been associated with a higher incidence of PTSD [26,29,79,80].

We hypothesize that as GCs reach a critical threshold level during PSS exposure, they function as an alarmin, inducing HMGB-1, thereby preparing an organism’s innate immune system. Inadequate GC release in a timely manner will thus cause dysregulation (dysfunction or overstimulation) of microglia activity and functions, resulting in disruptions in integrated circuit regulation (allostasis). Timing, concentration, and duration of GC exposure are therefore all critical in determining whether these hormones will transient activated microglia. Taken together, we hypothesize that dysregulation of endogenous circulating glucocorticoids at the time of exposure plays a causal role in the development of microglial homeostasis imbalance that underlies the behavioral symptoms of the PTSD phenotype.

### 3.5. High-Dose Hydrocortisone Immediately Post-Exposure Changed Expression of Microglia Morphology

To assess this hypothesis, the behavioral effects of glucocorticoid pharmacological manipulation using hydrocortisone (GC agonist) and RU486 (GC receptor antagonist) were examined. We demonstrate that early post-stressor intervention with hydrocortisone (25 mg/kg), which attenuates posttraumatic stress response, was related to a dramatic increase in the number and length of branches of hippocampal Iba-1 cells, as compared with the exposed group treated with saline. On the microglial morphological level, hydrocortisone treatment was associated with an increase in total branch number and length, alongside increased branches arborization and complexity, with significantly more branch intersections within each sphere at Sholl radii 5–30 mm as compared with the exposed group treated with saline. This may provide the infrastructural basis required for the observed attenuation of the physiological and behavioral stress responses. This pattern of response suggests that the single high-dose hydrocortisone treatment conferred some degree of resilience to subsequent trauma-related stress exposure.

In contrast, a single bolus of systemic treatment with RU486 was ineffective in attenuating stress-induced behavioral disruptions and significantly increased the propensity of individuals to develop extreme behavioral responses on day 7. A single treatment with RU486, injected immediately after stress exposure was associated with significantly poorer long-term outcomes and was associated with a chronic hyper-activated microglia phenotype.

**Figure 11:**
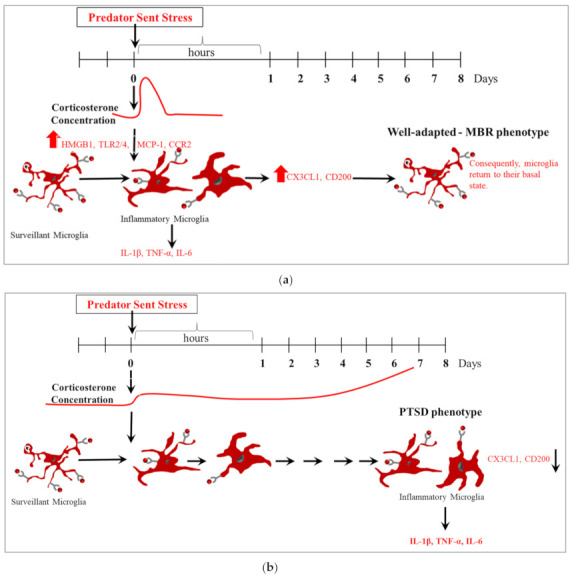
Either neuroprotective or neurotoxic microglial function may be involved at different stages of the development of PTSD phenotype. (**a**) Well-adapted (MBR) phenotype. (**b**) PTSD-phenotype.

Based on evidence that others and we have provided, we hypothesize that PSS may enhance microglial priming/activation as a protective mechanism (well-adapted (MBR) phenotype) (Figure 11a). Dysregulation of microglial activation or function during stress exposure may lead to an aberrant central immune response associated with behavioral stress disruption (PTSD phenotype) (Figure 11b). We further postulate that the beneficial (neuroprotective) or destructive (neurotoxic) outcome of the local microglial (innate) response to PSS will be determined, in part, by well-controlled transmission between the hypothalamic-pituitary-adrenal (HPA) axis and microglia signaling and/or neuronal-microglial communications. Therefore, we hypothesize that PSS-induced elevation of GCs initiates a cascade of events in the CNS leading to microglial proliferation: PSS exposure results in immediate HPA axis activation and elevations in endogenous GCs. GCs, via direct or indirect pathways, may act as a neuroendocrine danger signal, and initiate a process that sensitizes microglia [52]. We propose that as GCs reach a critical threshold during PSS exposure, they function as an alarmin, inducing HMGB-1, thereby preparing an organism’s innate immune system. Inadequate GC release in a timely manner will thus cause dysregulation (dysfunction or overstimulation) of microglial activity and function, resulting in disruption to integrated circuit regulation (allostasis). Timing, concentration, and duration of GC exposure are therefore all critical in determining whether these hormones will transiently activate microglia.

The above findings, although interesting, must be tempered by the limitations of the study: we must be sure to not be too literal in interpreting animal models and methods. It would be presumptuous (and spurious) to assume that the ‘criteria’ applied during this study, in fact, reflect psychophysiological parameters in the life of the rat, commensurate with the criteria for PTSD in humans.

To summarize, this study highlights the potential relevance of microglia to stress-related psychopathology and suggests a completely novel way forward in developing future therapeutics. Unraveling the psycho-neuro-immunological interplay in PTSD is an ongoing challenge that could result in effective therapies to prevent and treat PTSD.

## 4. Materials and Methods

All procedures were performed under strict compliance with ethical principles and guidelines of the National Institutes of Health (NIH) Guide for the Care and Use of Laboratory Animals. All treatment and testing procedures were approved by the Animal Care Committee of Ben-Gurion University of the Negev, Israel (IL-41-06-2019(C)).

### 4.1. Animals

One hundred and eleven adult male *Sprague Dawley* rats (Envigo RMS Israel Ltd., Jerusalem, Israel) weighing 200–250 g were habituated to housing conditions for a minimum of seven days. Animals were housed two per cage in a vivarium with stable temperature of 22 ± 1 °C and a 12 h light/dark cycle (lights on: 8:00 h) with unlimited access to food and water. All procedures were performed during the resting phase of the rats, between 07:30 and 18:30.

### 4.2. Experimental Design

To assess the role of microglia in the development of the PTSD phenotype in rats exposed to PSS, two experiments were conducted. Figure 1 and Figure 8 display the study design. In the first experiment (*n* = 55), forty-five animals were exposed to PSS and ten rats were exposed to sham-PSS (controls). Urine samples were collected 60–120 min following PSS exposure (see methods below). The behavioral assessments were conducted 7 days post-exposure, first in the elevated plus maze (EPM) paradigm and 1 h later in the acoustic startle reaction (ASR) paradigm. These data subsequently served for retrospective classification into behavioral response groups. The prevalence rates of extreme, partial, and minimal behavioral responses were assessed [2,5,6,7]. The rats were sacrificed one day later (Day 8) and their brains were collected for measurement of Iba-1 and CRXCR1 immunoreactivity (see methods below) in the dorsal hippocampus. The number of Iba-1 and CRXCR1 positive cells were evaluated from animals classified according to CBC’s. In the second study (*n* = 56), the long-term behavioral effects of hydrocortisone (25 mg/kg), RU486 (30 mg/kg) or saline injected 10 min after PSS exposure were evaluated using the EPM and the ASR paradigms 7 days post-exposure. To elucidate the molecular changes acutely coupled with these pharmacological manipulations, brains were collected at day 8 post exposure for measurement of Iba-1immunoreactivity.

### 4.3. Predator Scent Stress

Rats were individually placed on well-soiled cat litter, used for 2 days by a cat and sifted for stools [2,3,5,6,7,81]. Rats were exposed to the litter for 10 min in a plastic cage (inescapable exposure) placed on a yard’s paving stone in a closed environment.

Sham-PSS was administered under similar conditions, but rats were exposed to fresh, unused cat litter.

### 4.4. Drugs

Hydrocortisone (Sigma-Aldrich, Rehovot, Israel) in a dose of 25 mg/kg was dissolved in 9% saline solution. The Hydrocortisone dose was determined according to our previous study [82,83]. RU486 (Mifepristone) (Sigma-Aldrich, Israel) in a dose of 7.5 mg (approximately 30 mg/kg) was dissolved in 0.5 mL propylene glycol vehicle. Drug doses were chosen based on previous studies [84,85]. Saline (9%) solution in the same manner, controlling for potentially stressful effects of the IP administration. The drug/vehicle solutions were freshly prepared and injected at the same time of day in a volume of 1 mL/kg body weight.

### 4.5. Behavioral Assessments

All behavioral tests were performed in a closed, quiet, light-controlled room in the Faculty of Medicine, Anxiety and Stress Research Unit, Ben-Gurion University between 10:00 and 16:00 h. All behavioral tests were video-recorded for future analysis using the ETHO-VISION program (Noldus), by an investigator blinded to the experimental protocol.

#### 4.5.1. Elevated-Plus Maze (EPM)

The EPM consists of a plus-shaped platform with two open arms and two closed arms—surrounded by 14 cm high opaque walls on three sides, with arms of the same type located opposite each other. Procedure: Each rat was placed on the central platform facing an open arm and was allowed to explore the maze for 5 min. Each test was videotaped and recorded by an independent observer. Arm-entry was defined as entering the arm with all four paws. The following parameters were measured: duration in open and closed arms, and on the central platform; open and closed arm entries; and total entries into all arms (=total exploration).

#### 4.5.2. Acoustic Startle Response (ASR)

Startle response was measured using two ventilated startle chambers (SR-LAB system, San Diego Instruments, San Diego, CA, USA). The SR-LAB calibration unit was used routinely to ensure consistent stabilimeter sensitivity between test chambers and over time. Each Plexiglas cylinder rests on a platform inside a sound-proofed, ventilated chamber. Movement inside the tube is detected by a piezoelectric accelerometer below the frame. Sound levels within each test chamber are measured routinely using a sound level meter (Radio Shack) to ensure consistent presentation. Each test session started with a 5 min acclimatization period to background white noise of 68 dB, followed by 30 acoustic startle trial stimuli in six blocks (110 dB white noise of 40 ms duration with 30 or 45 s inter-trial interval). Behavioral assessment consisted of: mean startle amplitude (average over all 30 trials) and percent of startle habituation to repeated presentation of the acoustic pulse. Percent habituation—the percent change was calculated between the response to the first and last (6th) blocks of sound stimuli, as follows:

$$Percent\;Habituation=100\times \left[\frac{\left(average\;startle\;amplitude\;in\;Block\;1\right)+\left(average\;startle\;amplitude\;in\;Block\;6\right)}{average\;startle\;amplitude\;in\;Block\;1}\right]$$

### 4.6. Cut-Off Behavioral Criteria Model

Individual rats were classified according to the degrees to which individual behavior was affected by a stressor. The classification of individual rats was based on the premise that extremely compromised behavior in response to the priming trigger is not conducive to survival, inadequate and maladaptive, and thus represents a pathological degree of responses [2,3,5,6,7,81]. Please see Supplementary Material. 

### 4.7. Urine Corticosterone Sampling

Urine samples were collected for corticosterone levels by gently removing each rat to metabolic cages for 30 min, as described previously [29]. Animals were placed in these cages from their home cage and, once the procedure was complete, the animal was returned to its home cage. Rats were allowed to acclimate to the metabolic cage for 7 days before urine collection. Metabolic cages are regular cages with grooves along the floor, allowing for urine collection in suspended calibrated cylinders. All samples were immediately frozen (−80 °C) after collection. Samples were taken 60–90 min following exposure (between 12:30 and 14:00 h). According to the manufacturer’s instructions, corticosterone concentrations were measured with a DSL-10-81000 ELISA kit (Diagnostic Systems Laboratories, Webster, TX, USA) by a person blind to experimental procedures. All samples were measured in duplicate.

### 4.8. Immunohistochemistry

Tissue preparation: 3–5 min after completion of the protocol, animals were deeply anesthetized (ketamine and xylazine mixture) and perfused transcardially with cold 0.9% physiological saline followed by 4% paraformaldehyde (Sigma-Aldrich, Rehovot, Israel) in 0.1 M phosphate buffer (pH 7.4). Brains were quickly removed, post-fixed in the same fixative for 12 h at 4 °C, and cryoprotected overnight in 30% sucrose in 0.1 M phosphate buffer at 4 °C. Brains were frozen on dry ice and stored at −80 °C. Serial coronal sections (10 µm) at the level of dorsal hippocampus were collected from each animal using a cryostat (Leica CM 1850, Wetzlar, Germany) and mounted on coated slides.

#### 4.8.1. Staining

Iba-1 staining protocol: Sliced sections were air dried and were incubated for 120 min in a permeabilization solution: 0.3% TritonX-100 in PBS (PBS/T). After washes in PBS/T, the sections were incubated for 120 min in a blocking solution: 1% BSA and 0.3% PBS/T, and then were incubated overnight at 4 °C with primary antibody against Iba-1 (rabbit polyclonal anti-Iba-1 antiserum (1:10,000), product code: 019-19741, Fujifilm Wako Chemicals Europe GmbH, Neuss, Germany) 1:10,000 in 1% BSA + 0.3% PBS/T. After three washes in PBS/T, sections were incubated in DyLight-488 labeled goat-anti-rabbit IgG (1:10,000; Thermo Fisher Scientific Inc., Waltham, MA, USA) in 0.3% PBS/T Sections were washed and mounted with mounting medium (Vectrastain Vector laboratories, Burlingame, CA, USA). Control staining was performed in the absence of the primary antibodies.

CX3CR1 staining protocol: The sections dissected and processed by the CX3CR1 Rabbit kit (Abcepta, San Diego, CA, USA), according to the manufacturer’s instructions. On day 1, sections were washed 3 times (5 min each) in permeabilization: 0.3% TritonX-100 in PBS (Phosphate Buffered Saline). Brains left in blocking solution (1% Bovine serum albumin (BSA) + 0.3% TritonX-100 in PBS) for 2 h. Sections left in a primary antibody (Rabbit CX3CR1 1/250 in 1% BSA + 0.3% TritonX-100 in PBS) overnight at 4 °C in a humidified chamber. On day 2, the sections were washed 3 times (5 min each) in the same solution as Day 1. A secondary antibody administered: AlexaFlour 488 anti-rabbit IgG 1/400, 0.3% TritonX-100 in PBS. The sections rinsed 3 times (5 min each) in 0.3% TritonX-100 in PBS. Sections mounted with DAPI and covered with glass slides.

#### 4.8.2. Quantification

Each brain region was defined under the microscope according to cytoarchitectural landmarks [86]. Regions of interest were outlined, and computer-aided estimation was used to calculate the number of Iba-1-ir and CX3CR1-ir positive cells within the pyramidal layer of the CA 1 and CA3 and the granular layer of the DG of the hippocampus. Measurements occurred in a 50,000-mm^2^ area in each area of interest and were digitized using microscopic images (Leica microscope DM4500B; Leica Microsystems, Wetzlar, Germany) and a DFC340FX digital imaging camera (Leica Microsystems, Wetzlar, Germany). Measurements were taken in each sub-region from both brain hemispheres. The number of positive cells expressing Iba-1 and CX3CR1, were determined with the Leica LAS software (version 3.8). Sections from the brains of different groups of rats were processed at the same time and under identical conditions to ensure reliable comparisons and to maintain stringency in tissue preparation and staining conditions. Seven representative sections of the hippocampus were chosen (between Bregma −2.30 and Bregma −3.60) from each animal, from each group.

#### 4.8.3. Morphological Analysis of Microglia Cells

To reconstruct the glial morphology, a researcher blinded to the identity of the groups performed the morphological analysis using ImageJ software. The number and length (μm) of the Iba-1-positive cells and branches, along with the cell soma size, were measured using ImageJ software. Microglia were selected according to the following criteria: uniform labeling, without any reaction precipitate; complete staining of the glia body and its branches; relative isolation of the glia from the surrounding microglia to obtain a clear image of the entire cell; cell location in the CA1, CA3, and DG of the dorsal hippocampus.

#### 4.8.4. Region of Interest

The hippocampus was chosen as target in this study: The hippocampus has been demonstrated as particularly vulnerable to toxic effects of stress [87,88]; and show robust stress-induced priming of neuroinflammatory processes in vivo [42,89] and ex vivo [49].

### 4.9. Statistical Analyses

Data are presented as the mean ± S.E.M., unless otherwise specified. A *p* < 0.05 was statistically significant. For behavioral and molecular tests, statistical analyses were one-way analysis of variance (ANOVA). For the Sholl analysis, a two-way ANOVA was used. Post hoc Bonferroni tests were used to examine differences between individual groups. In addition, behavioral data were transformed to percentages using the cut-off behavioral criteria model: the prevalence of affected rats as a function of the rat group was tested by using cross-tabulation and nonparametric Chi-squared tests. All nonparametric analyses were performed on raw data (and not on percentage). Pearson’s correlation analysis was used to describe the relationship between Iba-1-ir cells and corticosterone levels across the entire sample.

## 5. Conclusions

The main findings of this study included: (1) Aberrant microglial homeostasis is involved in PSS-induced impairment in behavioral stress responses. The PTSD-phenotype group had a significantly higher cell density within the hippocampal subregions than in PBR, and MBR rats and compared with sham-PSS controls. Changes in microglial morphology have also been related only to the PTSD-phenotype group. The microglial cells within the hippocampus subregions showed decreased branching and increased nuclear area. Additionally, PTSD-phenotype group had a significantly lower CX3CR1 within the hippocampal subregions than in PBR, and MBR rats and compared with sham-PSS controls. Taken together, PSS shifts the state of microglia from a quiescent phenotype to a chronic activated phenotype only in PTSD phenotype rats. PSS may weaken inhibitory control over microglia, thereby permitting these cells to shift from a surveillant state to a primed state of activation. (2) Aberrant HPA axis activity is involved in PSS-induced impairment in microglia homeostasis and in behavioral responses: Dysregulation of endogenous circulating corticosterone at the time of exposure plays a causal role in the development of microglial homeostasis imbalance that underlies the behavioral symptoms of the PTSD phenotype. Early post-stressor treatment with high dose hydrocortisone (25 mg/kg), which attenuates posttraumatic stress response, was associated with a dramatic increase in the number and length of branches of hippocampal Iba-1 cells, as compared with the exposed group treated with saline. Single treatment with RU486, injected immediately after stress exposure were associated with significantly poorer long-term outcome and was associated with a chronic hyper-activated microglia phenotype.

## Figures and Tables

**Table 1:** Quantitative analysis of total microglia number total microglia length (mm), soma size and computer-generated plots of reconstructions of the branches tree in CA1 area in sham-PSS controls, animals with an extreme behavioral response (EBR) or partial response (PBR) to the PSS exposure, and in animals whose behavior was minimally affected by the stressor (MBR). All data represent the group means ± S.E.M. NS = Not significant.

	Control N = 8 I	EBR N = 10 II	PBR N = 11 III	MRB N = 10 IV	Statistics
CA1:					
Number of branches	11.25 ± 0.9	6.5 ± 0.85	7.5 ± 1.3	8.6 ± 0.8	I ≠ II
Branches Length	169.3 ± 12.6	97.8 ± 13.7	115.2 ± 14.3	130.3 ± 10.4	I ≠ II, III
Soma Area	30.1 ± 1.8	39.4 ± 2.9	28.6 ± 2.5	29.6 ± 2.4	II ≠ III, IV
CA1 reconstructions of the branches tree	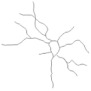	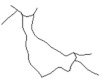	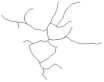	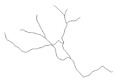	
CA3:					
Number of branches	15.4 ± 1.7	5.4 ± 0.7	11.2 ± 0.8	23.7 ± 1.4	II ≠ I, III, IV; IV ≠ I, III
Branches Length	210.9 ± 19.7	63.9 ± 9.2	166.9 ± 11.9	286.8 ± 13.7	II ≠ I, III, IV; IV ≠ I, III
Soma Area	31.3 ± 1.4	35.3 ± 3.8	32.4 ± 1.7	28.4 ± 1.9	NS
Reconstructions of the branches tree	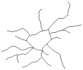	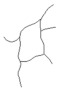	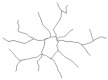	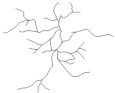	
DG:					
Number of branches	24.9 ± 1.9	10.0 ± 2.8	17.1 ± 1.3	14.8 ± 2.1	I ≠ II, IV
Branches Length	256.1 ± 17.0	130.7 ± 28.6	213.5 ± 15.8	188.6 ± 31.6	I ≠ II
Soma Area	25.1 ± 1.3	36.8 ± 3.0	31.4 ± 4.1	28.3 ± 2.4	NS
Reconstructions of the branches tree	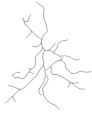	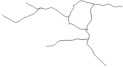	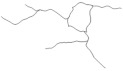	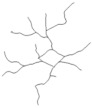	

**Table 2:** Effects of high-dose hydrocortisone, saline or RU486 early post-exposure on Iba-1 positive cell counts in hippocampal subregions. Results displayed as mean ± S.E.M. NS = Not significant.

	Sham-PSS N = 10 I	PSS + Saline N = 16 II	PSS + Hydrocortisone N = 16 III	PSS + RU486 N = 14 IV	Statistics
CA1: Number of branches ^1^ Branches Length ^2^ Soma Area ^3^	12.9 ± 1.0 185.85 ± 13.1 28.9 ± 1.7	7.8 ± 1.0 111.6 ± 11.9 35.3 ± 2.0	14.2 ± 0.7 190.4 ± 9.2 30.4 ± 1.4	8.4 ± 0.7 104.7 ± 5.2 36.1 ± 2.3	I, III ≠ II, IV I, III ≠ II, Iv I ≠ IV
CA3: Number of branches ^4^ Branches Length ^5^ Soma Area ^6^	17.1 ± 2.0 204.9 ± 19.5 30.5 ± 1.4	9.0 ± 1.0 124.6 ± 14.9 37.4 ± 2.0	17.0 ± 1.1 248.6 ± 10.9 30.7 ± 1.4	10 ± 1.0 125.5 ± 10.6 40.8 ± 2.1	I, III ≠ II, Iv I, III ≠ II, IV III≠II, IV; ≠IV
DG: Number of branches ^7^ Branches Length ^8^ Soma Area ^9^	22.1 ± 1.7 254.5 ± 16.1 27.8 ± 2.8	12.3 ± 1.6 153.9 ± 18.0 33.5 ± 2.9	18.6 ± 1.7 232.4 ± 18.1 26.1 ± 1.7	13.4 ± 1.3 140.7 ± 7.6 35.5 ± 2.9	I ≠ II, IV; II ≠ III I, III ≠ II, IV III ≠ IV

^1^ F(3, 52) = 14.4, *p* < 0.0001; ^2^ F(3, 52) = 21.0, *p* < 0.0001; ^3^ F(3, 52) = 3.3, *p* < 0.03; ^4^ F(3, 52) = 13.8, *p* < 0.0001; ^5^ F(3, 52) = 24.8, *p* < 0.0001; ^6^ F(3, 52) = 7.5, *p* < 0.0003; ^7^ F(3, 52) = 8.0, *p* < 0.0003; ^8^ F(3, 52) = 11.4, *p* < 0.0001; ^9^ F(3, 52) = 3.0, *p* < 0.04.

## Data Availability

Not applicable.

## Supplementary Materials

The following supporting information can be downloaded at: https://www.mdpi.com/article/10.3390/ijms23137185/s1.

## Abbreviations

ANOVA	Analysis of variance
CA	Cornu Ammonis
CNS	Central nervous system
CX3CR1	CX3C chemokine receptor 1
DG	Dentate gyrus
EBR	Extreme behavioral response
EPM	Elevated plus-maze
GC	Glucocorticoid
GR	Glucocorticoid receptor
HPA	Hypothalamus-pituitary-adrenal
Iba-1	Ionized calcium-binding adaptor molecule 1
IL-1β	Interleukin-1β
Ir	Immunoreactive
MBR	Minimal behavioral response
NF-κB	Nuclear factor-κB
PBR	Partial behavioral response
PSS	Predator scent stress
PTE	Potentially traumatic experience
PTSD	Posttraumatic stress disorder
TFs	Transcription factors
TNF-α	Tumor necrosis factor α
URA	Upstream regulator analysis
