# Supplementary material for: The Role of Microglia in the (Mal)adaptive Response to Traumatic Experience in an Animal Model of PTSD
Source: Int J Mol Sci. 2022 Jun 28;23(13):7185. doi: 10.3390/ijms23137185 (PMC9266429; doi:10.3390/ijms23137185)
Supplement: Supplementary file 1 [file ijms-23-07185-s001.zip › ijms-1745840-supplementary.pdf]

Supplementary Materials

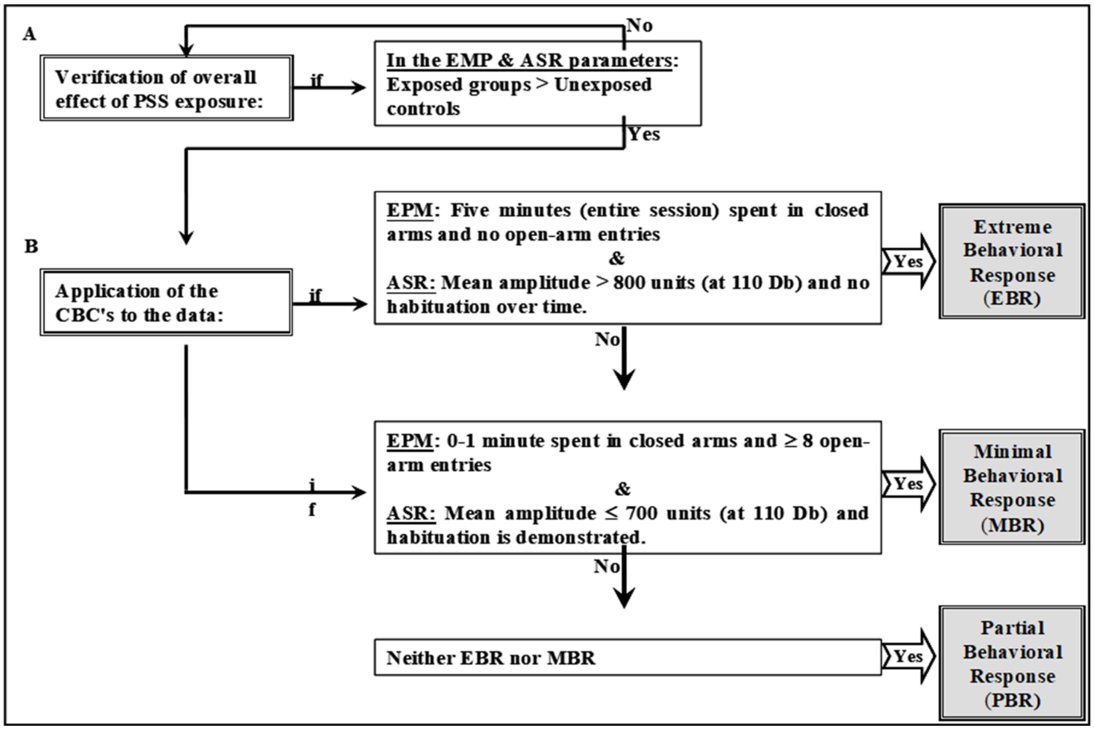

Figure S1. The cut-off behavioral criteria algorithm: To approximate the behavioral model to contemporary clinical conceptions of post-traumatic stress disorder (PTSD), we classified animals into groups according to degree of response to the stressor (PSS, predator scent stressor), i.e., the degree to which the behavior of an individual is altered or disrupted. To this end, behavioral criteria were defined and then complemented by the definition of cut-off criteria, which reflect the severity of response; this parallels inclusion and exclusion criteria applied in clinical research. The procedure requires the following steps: (A) Verification of global effect: the data must demonstrate that the stressor has a significant effect on the overall behavior of PSS- versus Sham PSS-exposed populations at the time of assessment; (B) Application of the cut-off behavioral criteria to the data: to maximize the resolution and minimize false positives, extreme responses to the stress in both the elevated plus-maze and acoustic startle response paradigms (performed sequentially) are required for "inclusion" into the extreme behavioral response (EBR) group. A negligible response in both paradigms is required for inclusion into the minimal behavioral response (MBR) group. Individuals that are not classified as having an EBR or an MBR are, by default, classified as having a partial behavioral response (PBR).
